# M-HIFU Inhibits Tumor Growth, Suppresses STAT3 Activity and Enhances Tumor Specific Immunity in a Transplant Tumor Model of Prostate Cancer

**DOI:** 10.1371/journal.pone.0041632

**Published:** 2012-07-24

**Authors:** Xiaoyi Huang, Fang Yuan, Meihua Liang, Hui-Wen Lo, Mari L. Shinohara, Cary Robertson, Pei Zhong

**Affiliations:** 1 Department of Mechanical Engineering and Materials Science, Duke University, Durham, North Carolina, United States of America; 2 Division of Surgical Sciences, Departments of Surgery, Duke University School of Medicine, Durham, North Carolina, United States of America; 3 Department of Immunology, Duke University Medical Center, Durham, North Carolina, United States of America; 4 Department of Molecular Genetics and Microbiology, Duke University Medical Center, Durham, North Carolina, United States of America; 5 Division of Urology, Departments of Surgery, Duke University Medical Center, Durham, North Carolina, United States of America; University of Tokyo, Japan

## Abstract

**Objective:**

In this study, we explored the use of mechanical high intensity focused ultrasound (M-HIFU) as a neo-adjuvant therapy prior to surgical resection of the primary tumor. We also investigated the role of signal transducer and activator of transcription 3 (STAT3) in M-HIFU elicited anti-tumor immune response using a transplant tumor model of prostate cancer.

**Methods:**

RM-9, a mouse prostate cancer cell line with constitutively activated STAT3, was inoculated subcutaneously in C57BL/6J mice. The tumor-bearing mice (with a maximum tumor diameter of 5∼6 mm) were treated by M-HIFU or sham exposure two days before surgical resection of the primary tumor. Following recovery, if no tumor recurrence was observed in 30 days, tumor rechallenge was performed. The growth of the rechallenged tumor, survival rate and anti-tumor immune response of the animal were evaluated.

**Results:**

No tumor recurrence and distant metastasis were observed in both treatment groups employing M-HIFU + surgery and surgery alone. However, compared to surgery alone, M-HIFU combined with surgery were found to significantly inhibit the growth of rechallenged tumors, down-regulate intra-tumoral STAT3 activities, increase cytotoxic T cells in spleens and tumor draining lymph nodes (TDLNs), and improve the host survival. Furthermore, M-HIFU combined with surgery was found to significantly decrease the level of immunosuppression with concomitantly increased number and activities of dendritic cells, compared to surgery alone.

**Conclusion:**

Our results demonstrate that M-HIFU can inhibit STAT3 activities, and when combined synergistically with surgery, may provide a novel and promising strategy for the treatment of prostate cancers.

## Introduction

Prostate cancer is the most common cancer in men with about 230,000 cases diagnosed annually in the United States [1]. Among various treatment strategies used clinically, prostectomy remains the first curative option for prostate cancer patients [2], [3]. However, surgery is not suitable for treating distant metastases and is thus often employed in concert with radiation and/or chemotherapy in patients with advanced prostate cancers. While radiation and chemotherapy following surgery is often helpful, they may not be applicable to all patients depending on cancer stage and patient tolerance. In addition, the presence of blood-prostate barrier hinders the delivery of chemotherapeutic agents to prostate cancer cells [4]. More importantly, the low specificity of radiation and chemotherapy in targeting cancerous cells often lead to significant side effects. Therefore, it is highly desirable to combine surgery with a locally targeted therapy, such as high intensity focused ultrasound (HIFU, also known as high-intensity therapeutic ultrasound, HITU) that is well-tolerated by patients, to eradicate the primary tumor while providing the added benefit in suppressing tumor recurrence and distant metastasis.

In the past decade, HIFU has emerged as a promising non-invasive treatment modality for localized benign and malignant solid tumors [5]. Extensive animal and clinical studies have demonstrated that HIFU is well suited for thermal ablation of localized early-stage tumors and for salvage therapy following prior treatment failure by radiation or chemotherapy [6]–[8]. By focusing a high intensity ultrasound beam on a well-defined region inside a patient, the converging acoustic energy can be absorbed by the target tumor tissue, resulting in a rapid temperature elevation above 65°C in a few seconds, leading to coagulative cell necrosis [9]–[11]. Moreover, when HIFU is used in a pulsed mode with low duty cycle (<2%) through a process known as mechanical HIFU (M-HIFU), it can produce non-thermal, mechanical damage of the tumor tissue through acoustic cavitation [12]. These attributes make HIFU an inherently non-invasive, non-ionizing therapy that is generally well tolerated by the patients. Unlike radiation and chemotherapy, HIFU treatment can be administrated repeatedly without increasing the risk of systemic toxicity [5].

In addition to thermal ablation of tumor tissues, HIFU has been shown to trigger an anti-tumor immune response both in mice carrying neuroblastoma [9] and in patients with late-stage solid tumors [13]. It has been suggested that HIFU-ablated tumor debris and residual tumor-associated antigens may stimulate dendritic cells (DCs) and subsequent T cell activation, leading to the development of anti-tumor immune responses [14], [15]. This hypothesis is supported by several clinical studies showing increased lymphocyte cytotoxicity, improved survival rate, and reduced tumor recurrence and distant metastasis [16].

Compared with conventional HIFU that relies primarily on thermal ablation, we have demonstrated previously that M-HIFU can induce stronger release of endogenous danger signals [17], enhance DC and cytotoxic T lymphocyte (CTL) activities [12] with reduced distant metastasis [18]. Despite these encouraging observations, the overall anti-tumor immune response elicited by HIFU is still insufficient, presumably because of the immune tolerance developed during tumorigenesis [19], in which signal transducer and activator of transcription 3 (STAT3) has been shown to play a critical role [20]–[22]. STAT3 is known to be activated by immunosuppressive cytokines such as IL-10, which in turn can further enhance the expression of IL-10. In addition, activated STAT3 (p-STAT3/Y705) inhibits the expression of pro-inflammatory cytokines such as IL-12 [23] and induces the expression of Foxp3, an essential transcription factor for regulatory T cells (Treg) development [24]. Although STAT3 has been shown to play a pivotal role in tumorigenesis and in immune tolerance, its role in M-HIFU elicited anti-tumoral responses has not been investigated.

In this study, we explored the strategy of using M-HIFU as a neo-adjuvant therapy before surgical resection of the primary prostate tumor to assess the effects of this novel combination therapy on tumor local recurrence and distant metastasis. In particular, we focused on the role of STAT3 in modulating HIFU-induced anti-tumor immune responses. Our results showed that, compared to surgery alone, the combination of M-HIFU and surgery could significantly inhibit tumor recurrence, suppress the growth of rechallenged tumors while improving survival rate. Moreover, these observations were corroborated by the reduced p-STAT3 activity with concomitantly increased anti-tumor response. Altogether, our results suggest that M-HIFU in combination with surgery may provide a new therapeutic option in the management of prostate cancer.

## Results

### M-HIFU Disintegrates RM-9 Tumor Tissues, Inhibits Tumor Growth, and Improves Host Survival

**Figure 1:**
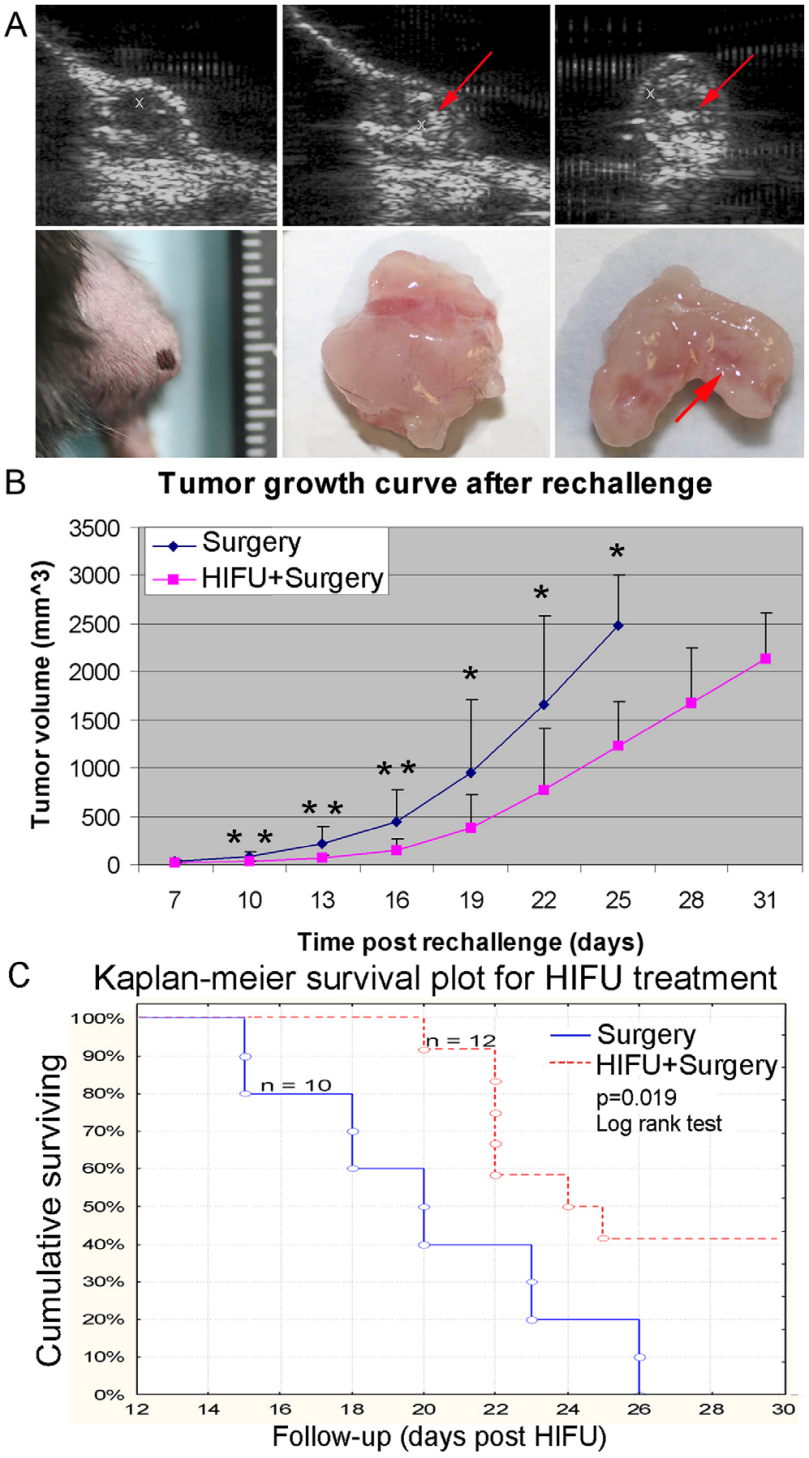
Therapeutic effects of M-HIFU on RM-9 tumors. *A,* Ultrasound imaging guided M-HIFU treatment. Cavitation activities induced by M-HIFU within the tumor tissue are shown in regions with hyperechogenicity, indicated by arrows in the B-mode images (upper panel) and in the excised tumors (lower panel. Left: skin on top of the tumor was intact after M-HIFU treatment; middle: removed tumor showing intact exterior surface after M-HIFU treatment; right: bisected tumor showing mechanical lesions in the interior region after M-HIFU treatment. *B*, Tumor growth curve post rechallenge. 2×10^4^ RM-9 cells were subcutaneously injected into the contralateral hind limb 30 days after M-HIFU treatment when the mice were fully recovered from surgical resection of the primary tumor. Volume of the rechallenged tumors was calculated based on caliper measurements of tumor size at 3-day intervals starting from day 7 following tumor rechallenge. *p<0.05, **p<0.01. *C,* Cumulative survival curve following tumor rechallenge. Mice reaching the humane endpoint were sacrificed. There were 10 mice in the surgery group and 12 in the HIFU + surgery group. P-value of 0.019 was determined by the log-rank test. Data were obtained from three independent experiments.

Figure 1A shows representative B-mode ultrasound images of the RM-9 tumor during M-HIFU treatment. The focus of the HIFU transducer was highlighted by a cross symbol. The M-HIFU treatment was started at the center and progressed with 1 mm step towards the tumor boundary. A safety margin of at least 1 mm was used during the HIFU treatment to avoid damage to adjacent skin, muscle and bone. Following each exposure, a bright spot of hyperechogenicity could be observed (indicated by arrows in the upper panels), corresponding to the production of inertial cavitation bubbles in the tumor tissue [12]. Because pulsed HIFU with a low duty cycle of 2% was used in M-HIFU, the resultant temperature within the tumor tissue was less than 45°C following a 20-s exposure. Therefore, thermal effect was negligible. Examination of the excised tumor tissues revealed disintegration of tumor tissue and formation of cavities that are characteristic of mechanical lysis produced by inertial bubbles in the HIFU treated region (lower right panel of Figure 1A).

Two days after M-HIFU treatment, the primary tumors were resected from the mice in both groups. This time delay was selected based on our previous observation that M-HIFU 2 days prior to surgical resection of the primary tumor can significantly reduce the risk of distant metastasis in a murine melanoma model [18]. Follow up observation in the next 30 days showed no local tumor recurrence, presumably as a result of the rigorous and comprehensive removal of tumor tissues by surgery. At this time, tumor rechallenge was performed and the growth of the rechallenged tumors was followed up for another 30 days. As shown in Figure 1B, there is a clear separation in the growth curves with the rechallenged tumors in the M-HIFU + surgery group significantly smaller than the ones in the surgery group. Significant differences in the tumor volume between the two groups were observed starting from day 10 until the end of the following up period.

Following rechallenge, no animals died. The mice with rechallenged tumors were only sacrificed when the humane endpoints for tumor growth were reached. Lung tissues were harvested and examined under a dissection microscope, but no metastatic nodule was observed. When surgery was performed, RM-9 tumor nodules were found to be well surrounded by a capsule of connective tissue, indicating a possible cause for the absence of distant metastasis. The cumulative survival in the M-HIFU + surgery group was found to be statistically higher than that in the surgery group (Figure 1C), indicating a potential tumor suppressing effect of M-HIFU in combination with surgical resection of the primary tumor.

### Prostate Cancer RM-9 Cells Constitutively Express p-STAT3, Which can be Reduced by M-HIFU Treatment

**Figure 2:**
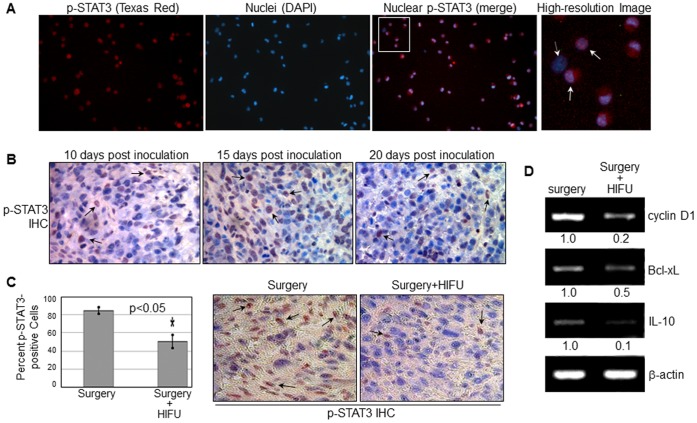
STAT3 is constitutively activated in RM-9 cells and tumors, and the level of activation can be suppressed by M-HIFU. *A,* Expression of p-STAT3 in RM-9 cells. RM-9 cells were subjected to IF staining for p-STAT3 and DAPI staining for nuclei. Under fluorescent microscopy, p-STAT3 was marked by red fluorescence while nuclei were labeled by blue signals. The p-STAT3-positive nuclei were indicated by merged pink signals. Solid arrows point to representative p-STAT3-positive nuclei whereas the dashed arrow marks a p-STAT3-negative nucleus. *B,* Expression pattern of p-STAT3 *in vivo.* RM-9 tumors were removed at three different developing time points as indicated and subjected to the IHC staining for p-STAT3. After counterstained by hematoxylin, p-STAT3-positive nuclei were indicated by dark brown signals (arrows) whereas p-STAT3-negative nuclei were in blue. *C,* Decreased p-STAT3 levels in RM-9 tumors after M-HIFU treatment. Two days after M-HIFU treatment, RM-9 tumors were removed and subjected to IHC staining for p-STAT3. Three tumors in each group were analyzed and the representative figures are shown. The IHC was independently performed thrice. Data was presented as Mean ± SD. The student’s *t*-test was conducted to determine p-values. *p<0.05. *D,* Expression down-regulation of three STAT3 downstream target genes by the M-HIFU treatment. Two days post M-HIFU treatment, RM-9 tumors was removed and mRNA was isolated. RT-PCR was then performed to detect levels of cyclin D1, Bcl-xL, IL-10 and β-actin. Band signals were quantified using the NIH ImageJ software. Representative images from three independent experiments are shown.

Phospho-STAT3 (Y705), the activated form of STAT3, plays a key role in eliciting immune surveillance, tumor development and tumor metastasis [20], [22], [25]. Therefore, inhibiting or demolishing active STAT3 is of interest for both direct anti-tumor treatment and tumor specific immunotherapy. Before elucidating the role of M-HIFU on STAT3 activation, we examined several tumor cell lines for STAT3 activity and found that a high level of p-STAT3 was expressed in RM-9 cells. As shown by IF staining and fluorescence microscopy (Figure 2A), nearly all of the RM-9 cells were stained positively for p-STAT3, as indicated by the pink merged signals (arrows). This *in vitro* observation was further confirmed *in vivo*. As shown in Figure 2B, we examined RM-9 tumors harvested at three different time points (10-, 15-, and 20-days) post inoculation for p-STAT3 using IHC and the results indicate constitutive STAT3 activation in RM-9 tumors over the 20 days of tumor development. These observations also indicate that RM-9 cells and tumors are ideal for examining the effects of M-HIFU on STAT3 activity.

Next, we evaluated the effects of M-HIFU on STAT3 activation using RM-9 tumor-bearing mice. Two days after M-HIFU treatment, RM-9 tumors were removed from the mice and subjected to p-STAT3 IHC (n = 3 per group). As demonstrated in Figure 2C, p-STAT3 level was significantly decreased by 1.8-fold following M-HIFU, indicating that administration of M-HIFU can efficiently inhibit STAT3 activation in RM-9 tumors. Furthermore, consistent with the observed reduction of p-STAT3 by M-HIFU, expression levels of three STAT3 target genes were significantly reduced by M-HIFU as well, as indicated by the results of RT-PCR in Figure 2D. Altogether, these results demonstrate that STAT3 activation can be detected in RM-9 prostate cancer cells *in vitro* and *in vivo,* and that M-HIFU reduces the levels of STAT3 activation and STAT3-targeted genes in RM-9 tumors.

### M-HIFU Boosts Tumor-specific Cytotoxicity

**Figure 3:**
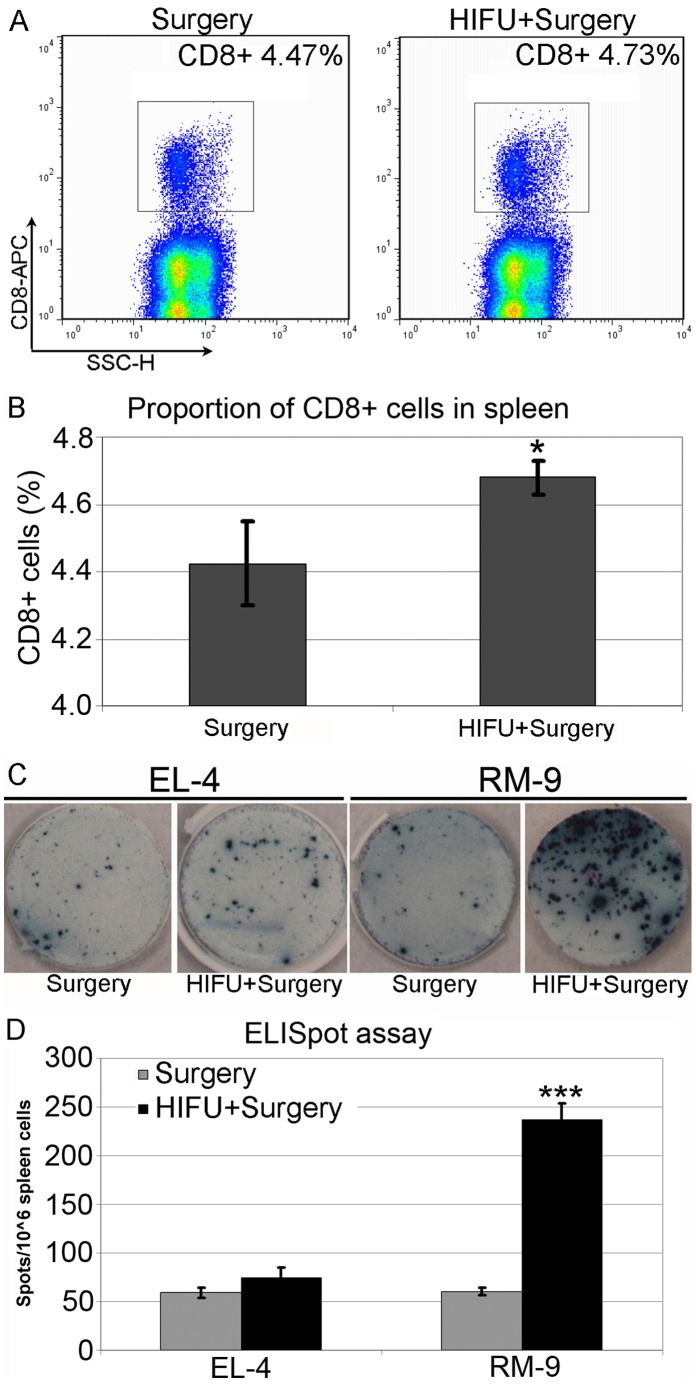
Increased anti-tumor T cell responses after M-HIFU treatment. *A,* 2 days after treatment, spleens from three mice treated with or without M-HIFU were collected and pooled. Proportion of splenic CD8^+^ T cells were determined by flow cytometry. Representative results from three independent experiments are shown. *B,* Statistical significance of the increase of the CD8^+^ cell population by combining HIFU with surgery. *p<0.05 *C*, Twelve days after surgery or M-HIFU plus surgery treatment, three mice from each group were sacrificed and their spleens were collected and pooled. IFN-γ ELISpot assay was performed with the pooled splenocytes. Mitomycin-C treated RM-9 cells were added as stimulators at the ratio of 1∶10 (RM9:splenocytes). EL-4 cells were used as an irrelevant cell control. Representative results from three independent experiments are shown. *D*, Enumeration of IFN-γ-positive cells and, and statistical analysis based on the ELISpot assay results. ***p<0.0001.

To investigate whether M-HIFU treatment results in the increase of CTL responses [26], we analyzed alteration in the frequency of CD8^+^ T cells and their IFN-γ production, both in the M-HIFU + surgery group and in the surgery alone group. We found that 2 days post surgery the number of CD8^+^ T cell was increased in the M-HIFU + surgery group (Figure 3A and B), compared to the group that had surgery alone. Tumor-specific IFN-γ production in the tumor-bearing hosts was further verified by ELISpot assay. Splenocytes were harvested 12 days post surgery from three mice per group, and the samples were pooled and co-cultured with either RM-9 or irrelevant EL-4 tumor cells (splenocytes vs. tumor cells at 10∶1 ratio), then analyzed to determine IFN-γ production. M-HIFU treatment significantly increased IFN-γ producing CTL to 4 fold of the non-HIFU control (p<0.001) (Figure 3C, D). This elevated response was not observed in EL-4 cells, indicating that the IFN-γ production was RM-9 tumor specific by the tumor-bearing host. These results suggest that M-HIFU treatment can promote generation of IFN-γ producing CTL, one of the most critical factors in anti-tumor immunity.

### M-HIFU Treatment Increases the Population of CTLs and DCs in Spleens and TDLNs

**Figure 4:**
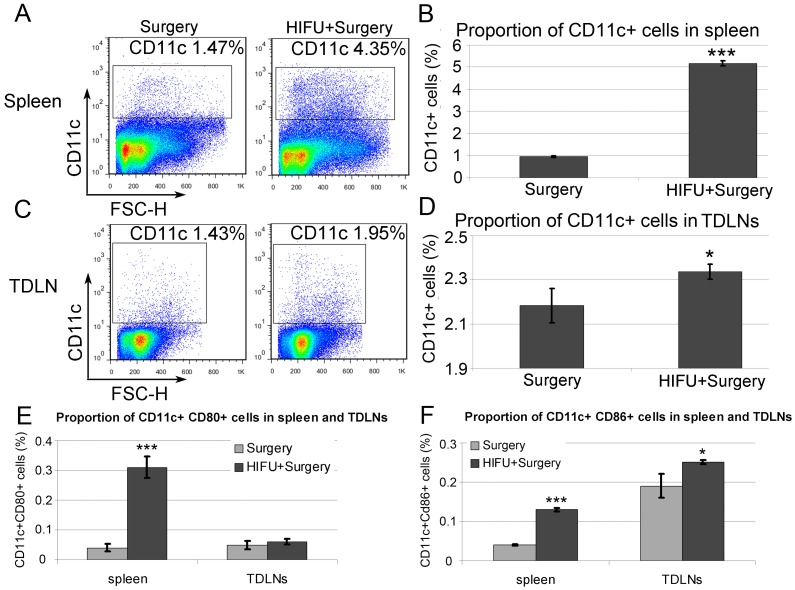
Increased DC population after M-HIFU treatment. Three mice from each group were sacrificed six days after surgery or HIFU plus surgery treatment. Single cell suspension was prepared from the pooled spleens or TDLNs; and CD11c^+^ cell population was determined by flow cytometry. Representative results from three independent experiments are shown. *A*, *C*, Representative flow patterns in detecting CD11c^+^ cells in spleen (*A*) and TDLNs (*C*). *B*, *D*, Statistical significance in the increase of the DC population in spleen (*B*) and TDLNs (*D*) based on the results of *A* and *C,* respectively. *E*, *F*, Evaluation of DC stimulation by detecting CD80-positive (*E*) and CD86-positive (*F*) CD11c^+^ cells in spleen and TDLNs. *p<0.05, ***p<0.0001.

DCs are the only professional antigen-presenting cells that can prime naïve T cells, and plays a critical role in the generation of tumor-specific CTLs. DCs can be identified with the expression of CD11c as a cell surface marker. Since p-STAT3 is a pivotal suppressor of DC activation [27], [28], we evaluated the frequency of CD11c^+^ cell population in spleens and TDLNs 6 days post M-HIFU treatment in mice bearing RM-9 transplanted tumors. As shown in Figure 4A to 4D, proportions of CD11c^+^ cells were significantly increased in spleens and TDLNs to more than 3-fold and 1.2-fold of the control group without HIFU treatment, respectively (in both cases, p<0.001). We also evaluated the proportion of CD11c^+^ cells at the time point of 2 days after M-HIFU treatment, but it appeared to be too early to observe an increase in the DC population (data not shown). In addition, M-HIFU treatment enhanced the expression of co-stimulatory molecule, CD80 and CD86, on DCs (Figure 4E and 4F). Collectively, M-HIFU treatment appears to enhance recruitment and/or expansion of DCs, in addition to DC maturation.

### M-HIFU Enhanced Anti-tumor Response is, in Part, Caused by the Suppression of Treg Cell Generation

**Figure 5:**
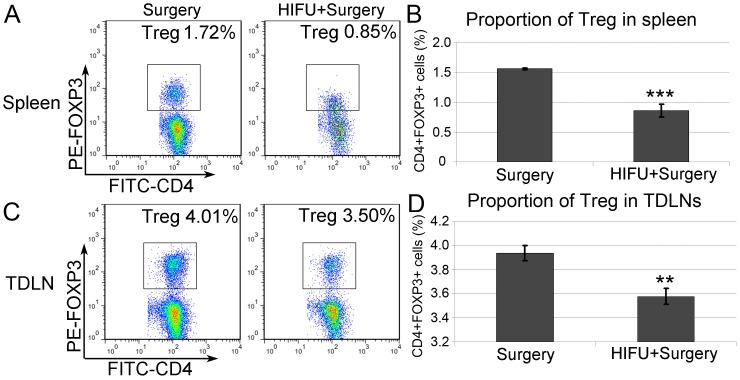
Decreased proportion of Treg after M-HIFU treatment. Single cell suspension was prepared from the pooled spleens or TDLNs. Population of CD4^+^ Foxp3^+^ cells was thereafter determined as Treg by flow cytometry. Figures showed are the representatives from three independent staining and subsequent flow analysis. *A*, *C*, Representative flow patterns in detecting Tregs in spleen (*A*) and TDLNs (*C*). *B*, *D*, Proportion of the Treg population in spleen (*B*) and TDLN (*D*) based on the results shown in *A* and *C*, respectively. **p<0.01, ***p<0.0001.

STAT3 also plays an important role in enhancing the development of CD4^+^ regulatory T cells (Tregs) [24]. Tregs inhibit immune responses and have been implicated in inducing tolerance in anti-tumor immunity. Here, we sought to determine whether M-HIFU treatment suppresses generation or expansion of Tregs in tumor-transplanted mice. Splenocytes and TDLNs cells were harvested 6 days after M-HIFU treatment as previously mentioned to evaluate the frequency of Tregs. The result shown in Figure 5 revealed that M-HIFU treatment elicited a substantial decrease in the Treg population by about 50% in spleen (p<0.001), and about 10% in TDLNs (p<0.001). Similar to the observation from DCs, changes in the Treg population were not significantly 2 days following M-HIFU treatment (data not shown). These results indicate that, in addition to expanding the DC population and increasing DC maturation, M-HIFU treatment reduced population size of Tregs, which are known to down-regulate the development of anti-tumor immunity.

## Discussion

This study was carried out to gain a better understanding of the mechanisms by which M-HIFU elicits anti-tumor effects and to explore strategies for the optimal use of M-HIFU in prostate cancer treatment. Our long-term goal is to establish rationales for using M-HIFU based combination therapy as an improved treatment modality for prostate cancer patients. Overall, our results suggest that M-HIFU can inhibit STAT3 activation, which leads to an anti-tumor response through two potential mechanisms. First, reduction of p-STAT3 in the tumors directly inhibits tumor growth. Second, a reduction in STAT3 activity leads to an increased number of tumor-specific CTLs and DCs, as well as a reduced number of Treg, and thereafter an enhancement of the anti-tumor immunity.

Constitutive activation of STAT3 is frequently observed in many human tumors, including melanoma, breast cancer, head and neck cancer, glioma and prostate cancer [20], [22], [25]. It is well known that persistently activated STAT3 functions as a key regulator of molecular and biological events that promote tumorigenesis [29]. Aberrant STAT3 activity plays a central role in dysregulated growth and survival of tumor cells as well as in promoting angiogenesis. Moreover, aberrant STAT3 promotes invasion and metastasis, thereby contributing to tumor progression [20], [22], [30]. During the course of tumorigenesis, the activity of STAT3 is also elevated in tumor-associated lymphocytes [31], [32]. Inhibiting STAT3 in either tumor cells or tumor-infiltrating myeloid cells elicits Th1 anti-tumor immune responses, concomitant with an increased number of CTLs and decreased Tregs [33]. Thus, suppressing the activation of STAT3 by physical (*i.e.,* HIFU) or pharmacological approaches is of great interest for both direct anti-tumor treatment and tumor specific immunotherapy.

Based on the aforementioned understanding about STAT3, we sought to determine the effect of M-HIFU on STAT3 activation in an *in vivo* transplant model of prostate cancer. Indeed, the level of intratumoral p-STAT3 was substantially decreased by the treatment of M-HIFU. In a previous study of B16 melanoma transplant model, p-STAT3 level was likewise found to be significantly down-regulated by M-HIFU treatment [34], suggesting the effects of M-HIFU on STAT3 activation may be common amongst different tumors.

The functional down-regulation of p-STAT3 was further verified by the decreased expression of its downstream target genes. Cyclin D1, which is up-regulated by activated STAT3, is an allosteric regulator of cyclin-dependent kinases 4 and 6 (CDK4/6) and promotes G1/S transition through the CDK4/6-mediated phosphorylation and inactivation of the retinoblastoma protein [32], [35]. Inhibiting the expression of cyclin D1 will retard the cell cycle in G1 phase from entering into S phase, and therefore, significantly decrease tumor cell proliferation [36]–[40]. Bcl-xL, also a major STAT3 target gene, is an anti-apoptotic molecule of the Bcl-2 family and enhances tumor progression [32], [41], [42]. Bcl-xL overexpression is frequently found in a wide spectrum of cancers including prostate cancer [43], [44]. Depletion of the predominant Bcl-xL expression significantly reduces the viability of tumor cells following apoptotic stimuli [45]. In this study, our results from RT-PCR clearly demonstrated that the down-regulation of p-STAT3 was accompanied by the decreased expression of cyclin D1 and Bcl-xL in tumor tissue following M-HIFU treatment. These results indicated that M-HIFU is capable of inhibiting the aberrant STAT3 activity and thereby may induce growth arrest and apoptosis of tumor cells, as well as regression of prostate cancer *in vivo*.

In addition to the intratumoral oncogenic role, p-STAT3 is involved in immune suppression through interfering DC stimulation by inhibiting the expression of proinflammatory cytokines and chemokines that stimulate DC [28]. Alternatively, p-STAT3 promotes tumor cells to produce multiple oncogenic cytokines, such as IL-10, which in turn activates STAT3 signaling through a positive feedback loop and inhibits functional DC stimulation [28], [32], [33]. DC stimulation is essential for effective antigen presentation and development of anti-tumor immune response by T cells. Our previous studies using the MC38 tumor model have shown that HIFU-generated mechanical damage of tumor cells can lead to DC stimulation [17], and subsequently increased CTL activities [12]. In this study, we further verified that M-HIFU can boost the function of DCs *in vivo*, as evidenced by the significant increased population of DCs with concomitantly upregulated expression of co-stimulatory molecules such as CD80 and CD86.

Another immune suppressive function of p-STAT3 is related to its role in enhancing the generation of Tregs, which suppress immune responses to self-antigen including tumor associated antigen. STAT3 also plays an important role by orchestrating multiple critical aspects of T cell function in inflammation and homeostasis. In particular, the expression of Foxp3, which is essential for the regulatory functions of Treg, depends on STAT3 activation [24]. Inhibiting STAT3 activity reduces conversion of Foxp3-negative T cells to tumor-associated Tregs and increases CD8^+^ T cell infiltration in tumor - a strategy that has been used to inhibit primary tumor growth [46]. Similarly, in our study, M-HIFU treatment was found to significantly decrease the proportion of Treg in both spleen and TDLNs, concomitantly with the suppression of STAT3 activity in those tissues. Therefore, our results suggest that M-HIFU can reduce Treg-elicited immune tolerance in the RM-9 model.

In summary, we have revealed a specific mechanism for M-HIFU induced anti-tumor immune response, which is mediated through inhibition of STAT3 activation *in vivo*. Further, we have found that M-HIFU performed at an appropriate time (i.e., 2 days for RM-9 model) prior to surgical removal of the primary tumor can enhance protection of the host against tumor recurrence, and therefore may increase the survival of patients from the malignancy of prostate cancer. Although in this study we focused on the mechanism associated with STAT3, involvement of other mechanisms for M-HIFU induced anti-tumor immunity is also possible, and will be investigated in the future. Altogether, we have demonstrated that M-HIFU is a promising neo-adjuvant therapy that may be combined synergistically with surgery for prostate cancer treatment.

## Materials and Methods

### Animal Model

C57BL/6J mice, 6∼8 weeks old, were purchased from the Jackson Laboratory (Bar Harbor, ME, USA) and housed in the Duke Vivarium before and after M-HIFU treatment and/or surgery. All the animals were handled in accordance with the established animal care policy and all animal experiments were approved by the Duke University Institutional Animal Care & Use Committee.

### Cell Line and Cell Culture

Murine prostate cancer cell line RM-9, established by Dr. Thompson in Baylor College of Medicine, was a gift from Dr. Mouraviev. EL4 mouse lymphoma cell line was purchased from American Type Culture Collection (ATCC, Manassas, VA, USA). All the cell lines were maintained in complete Dulbecco’s modified eagle medium (DMEM, Invitrogen, Carlsbad, CA, USA), supplemented with 10% fetal bovine serum (Hyclone, Logan, UT, USA), 2 mM L-glutamine, 50 IU/mL penicillin and 50 µg/mL streptomycin (Invitrogen, Carlsbad, CA, USA) at 37°C with 5% CO_2_.

### Antibodies, Kits and Chemical Reagents

The following kit and antibodies were purchased from eBiosciences (San Diego, CA, USA): Mouse regulatory T cell Staining Set (FJK-16 s), PE-conjugated anti-mouse CD8 antibody, and anti-mouse CD16/32 antibody. APC-conjugated anti-mouse CD11c antibody, FITC-conjugated anti-mouse CD80, and PE-conjugated anti-mouse CD86 were from BD bioscience (San Jose, CA, USA). Rabbit anti-mouse phospho-STAT3 (Y705) antibody was purchased from Cell Signaling (Danvers, MA, USA). Mouse Interferon-γ ELISpot kit was from MABTECH Inc (Cincinnati, OH, USA). Texas Red-conjugated goat anti-rabbit antibody was purchased from Vector Laboratories Inc (Burlingame, CA, USA). Mounting medium solution with or without DAPI, blocking solution, VECTASTAIN Elite ABC Kit (Rabbit IgG) and AEC kit were from Vector Lab. SV total RNA isolation system was purchased from Promega (Madison, WI, USA). DNA synthesis enzymes were from Invitrogen unless otherwise indicated.

### Tumor Model, HIFU Treatment, Surgical Resection, and Post-operative Care and Measurements

RM-9 tumor model was established by subcutaneously injecting 8×10^4^ RM-9 cells into the right hind limb of C57BL/6J mice. Tumors were allowed to grow for about 7 days until they reached 5∼6 mm in diameter. At this time, the mice were randomly allocated to two groups: surgery alone and M-HIFU + surgery. After anesthetized via IP injection of pentobarbital (50 mg/kg), each animal was treated either by M-HIFU using our established protocol [12], [18] or received sham HIFU exposure. Briefly, the HIFU transducer was operated at 3.3 MHz in pulsed mode for 20 s with a duty cycle of 2%, corresponding to a peak positive/negative pressure of 32/−10 MPa with an acoustic power of 60 W [47]. Under this exposure condition, the temperature elevation in tumor tissue near the HIFU focus was found to be less than 45°C. B-mode ultrasound imaging was performed during HIFU exposure to ascertain the production of inertial cavitation in the tumor tissue at each treatment spot [12]. HIFU treatment was started from the center of the tumor nodule and expanded gradually towards the boundary with a step size of 1.0 mm. A safety margin of 1 mm was used during the HIFU treatment to avoid damage to adjacent skin, muscle and bone. Depending on the tumor size, a total of 10 to 15 points were treated.

Two days following HIFU or sham treatment, the primary tumor was removed surgically using a resection margin of at least 2 mm into the surrounding soft tissues. Skin above the tumor tissue was also removed. Afterwards, the incision site was closed with absorbable sutures. Excised tumor tissues were retained for immunohistochemistry analysis.

When fully recovered, the animals were sent back to Vivarium and observed for 4 weeks or until the humane endpoints defined by the Duke Tumor Policy (i.e., overall tumor volume exceeding 2×10^3^ mm^3^ or ulceration of tumor) had reached. Tumor recurrence and survival rate were monitored during this observation period.

Two, six or twelve days post M-HIFU treatment, three mice in each group were randomly euthanized and the spleens and tumor draining lymph nodes (TDLNs) were harvested and pooled for flow cytometry or ELISpot assay.

Animals were considered to be completely recovered and free of recurrence when no local tumor growth was observed four weeks after surgery [48]. Rechallenge was performed 30 days after surgical resection of the primary tumor if no local recurrence had been observed. A total of 5×10^3^ RM-9 cells were subcutaneously injected into the contralateral limb of the mouse. The animal was then followed up for another 30 days, during which the recurrent tumor volume and survival rate were determined.

### Flow Cytometry

Cell staining with antibodies and flow cytometry analysis were performed as previously described [49], [50]. Briefly, single cell suspension from spleens or TDLNs (1×10^6^/staining) was prepared and FcγR was blocked for 15 min by CD16/32 antibody (Fc Block) before cell surface staining. Followed by the cell surface staining, intracellular staining of Foxp3 to identify Tregs was performed with fixation/permeabilization working solution (eBioscience). Tregs and DCs were identified by CD4^+^Foxp3^+^ marker and CD11c^+^ marker, respectively, and processed with a FASCalibur flow cytometer (BD, Mountain View, CA, USA). Flow data was analyzed with FlowJo software (Ashland, OR, USA).

### RNA Isolation and RT-PCR

Tumor tissues with or without M-HIFU treatment were homogenized before total RNA was isolated. Then reverse transcription and PCR was performed as described previously [51]. PCR reaction conditions were 1 cycle of 94°C for 10 minutes, followed by 21–25 cycles of 30 seconds at 94°C, 40 seconds at 55°C for amplification of cyclin D1, Bcl-xL and β-actin (57°C for IL-10), and 30 seconds at 72°C. The forward and reverse primers were 5′-ATCTAACATCCCAGCTTCACAT-3′ and 5′-GGCTGAAGAGAGAGTTGTGGT-3′ (Bcl-xL); 5′-GGACAACATACTGCTAAC CGACT-3′ and 5′-GAATAAATAGAATGGGAACTGAGGT-3′ (IL-10); 5′-CAGAAGTGCGA AGAGGAGGT-3′ and 5′-CGGTAGCAGGAGAGGAAGTT-3′ (cyclin D1); and 5′-GTCCCTCACCCTCCCAAAAG-3′ and 5′-GCTGCCTCAACACCT CAACC-3′ (β-actin).

### Immunofluorescent (IF) Staining for p-STAT3 (Y705)

This was conducted as previously described [52]. RM-9 cells in single suspension were fixed with 4% paraformaldehyde for 15 min at room temperature. After washing with cold PBS thrice, the cells were transferred to a Cytofunnel sample chamber (Thermo, Rockford, IL, USA) equipped with a glass slide and spun down with Shandon Cytospin centrifuge (Thermo). The cells were treated with PBS containing 0.2% Triton for 5 min at room temperature, washed with PBS and immerged into blocking solution containing PBS with 1% BSA and 5% normal goat serum for 1 hour in a humidified box. Following incubation with rabbit anti-mouse p-STAT3 antibody (1∶100; Cell Signaling) overnight at 4°C, cells were washed and incubated with a goat anti-rabbit antibody conjugated with Texas red (1∶200; Vector Lab) for 1 hour. The staining was terminated by washing the slide with PBS for three times. The slides were then mounted with DAPI containing VECTASHIELD mounting medium and examined under a fluorescent microscope (Carl Zeiss).

### Immunohistochemistry (IHC) For p-STAT3

As previously described [53], tumor tissues were collected from both treatment groups at indicated time and fixed overnight with 10% formalin. After being embedded in paraffin, tissues were cut at 5 µm thickness and mounted to glass slides. The tissue sections were deparaffinized, dehydrated, and subjected to antigen retrieval in an EDTA-containing buffer in an oven. Endogenous peroxidase activity was blocked with 0.3% hydrogen peroxide and the slides incubated with 10% normal goat serum for 30 min and then with rabbit anti-mouse p-STAT3 antibody (Cell Signaling; 9131S) at 4°C overnight. Following washes with PBS, the slides were incubated with biotinylated secondary antibodies and then with avidin-biotin-horseradish peroxidase complex. Detection was performed using 0.125% aminoethylcarbazole chromogen. After counterstaining with Mayer’s hematoxylin (Sigma), the slides were mounted. Scoring was conducted by evaluating at least 10 different fields per sample and three independent observations by different pathologists.

### ELISpot Assay

In order to assess anti-tumor cytotoxicity of CTLs, IFN-γ ELISpot assay was performed with the splenocytes twelve days after HIFU treatment as described before [12]. Briefly, ELISpot plate was coated overnight with the capture antibody (AN18) in sterile PBS. The plates were blocked for 30 min at room temperature with medium containing 10% FBS and washed 3 times with PBS. 1×10^6^ splenocytes from mice in each group were then plated and combined with either mitomycin-treated RM-9 or EL4 tumor cells, which serve as a negative control of the CTLs obtained from RM-9 transplanted mice at 10∶1 responder-to-stimulator ratio into the wells. After incubation for 36 h at 37°C and 5% CO_2_, the plate was washed with PBS, diluted detection antibody (R4-6A2-biotin) was added and incubated for 2 hours at room temperature, flowed by streptavidin-HRP for 1 hour. The spots corresponding to cytokine-producing cell colonies were visualized by incubation with 100 µl per well of TMB substrate solution and developed until distinct blue spots emerged at room temperature. IFN-γ secreting cell colonies were counted with the use of a computer-assisted video image analyzer (KS Elispot, Zeiss, Goettingen, Germany). The results were expressed as the number of spot-forming cells per 10^6^ input cells. Three independent experiments were performed with three duplicated wells included within each group.

### Statistical Analysis

Comparison between the two groups was carried out mostly by using the Student’s *t*-test with the results expressed as mean ± SD. The cumulative survival rate was calculated by using the Kaplan-Meier method and difference in the survival rate was evaluated by the log-rank test using Softstat software. P<0.05 was considered to be statistically significant.
